# ﻿A new species of *Otostigmus* (Chilopoda, Scolopendromorpha, Scolopendridae) from China, with remarks on the phylogenetic relationships of *Otostigmuspolitus* Karsch, 1881

**DOI:** 10.3897/zookeys.1168.82750

**Published:** 2023-06-29

**Authors:** Tian-Yun Chen, Chao Jiang, Lu-Qi Huang

**Affiliations:** 1 Guangdong Pharmaceutical University, Guangzhou 510006, China China Academy of Chinese Medical Sciences Beijing China; 2 State Key Laboratory of Dao–di Herbs Breeding Base, National Resource Center for Chinese Materia Medica, China Academy of Chinese Medical Sciences, Beijing 100700, China Guangdong Pharmaceutical University Guangzhou China

**Keywords:** Centipedes, key, phylogeny, *politus* species group, Yunnan-Guizhou plateau

## Abstract

Through a combination of morphological and DNA data, a new scolopendrid centipede from southern and southwestern China was revealed: *O.tricarinatus***sp. nov.** The species belong to the *politus* group but has three sharp tergal keels. Validation of phylogenetic status was performed through molecular analysis of the cytochrome *c* oxidase subunit I (COI), 16S rRNA, and 28S rRNA sequences from 16 *Otostigmus* species. *Otostigmustricarinatus***sp. nov.** was found to be two populations and varied in the number of spines on the ultimate prefemur, the sutures on a sternite, and a pore-free median longitudinal strip in the pore field. The Yunnan-Guizhou plateau population of *O.tricarinatus***sp. nov.** was sister to the clade *O.polituspolitus* + *O.politusyunnanensis* + Guangxi population of *O.tricarinatus***sp. nov.** with strong support from both BI (bayesian inference) and ML (maximum likelihood) analyses (PP = 1, BS = 97%).

## ﻿Introduction

The largest genus in the subfamily Otostigminae (Pocock, 1891), *Otostigmus* Porat, 1876, has approximately 110 recognised species. *Otostigmus* is classified into three subgenera: O. (Otostigmus), O. (Parotostigmus) Pocock, 1896, and O. (Dactylotergitius) Verhoeff, 1937 (Schileyko et al. 2020). Lewis (2010) arranged the subgenus O. (Otostigmus) into nine species groups that were based on the following characteristics: the presence or absence of keels on tergites, the number of antennal articles, the number of antennal articles which lacked numerous setae, sternite tuberculation, the number of tarsal spurs on legs, the ultimate leg prefemur characteristics and the coxopleural process of leg-bearing segment 21 with or without a dorsal spine.

Eight species have been recorded in China (Lewis 2003; Song et al. 2005; Niu et al. 2021), all assigned to the subgenus Otostigmus. These species belong to four of the nine groups mentioned by Lewis (2010):

1. the *politus* group (including *O.p.politus* Karsch, 1881; *O.p.yunnanensis* Lewis, 2003: antennae with 17 or 18 articles, 3 antennal articles glabrous, i.e., lacking numerous setae dorsally, the ultimate leg prefemur with one row of ventrolateral spines, the coxopleural process of leg-bearing segment 21 without a dorsal spine);
2. the *aculeatus* group (*O.aculeatus* Haase, 1881: antennae with 17 articles, 3 antennal articles glabrous dorsally, the ultimate prefemur with two rows of ventrolateral spines, the coxopleural process of leg-bearing segment 21 without a dorsal spine);
3. the *rugulosus* group (*O.astenus* Kohlrausch, 1881; *O.martensi* Lewis, 1992; *O.beroni* Lewis, 2001; *O.lewisi*Song et al., 2005; *O.xizangensis* (Niu et al. 2021): antennae with 18 articles, 2–2.75 antennal articles glabrous dorsally, coxopleural process of leg-bearing segment 21 with a dorsal spine);
4. the *scaber* group (*O.scaber* Porat, 1876: antennae with 21 articles, 2–2.5 antennal articles glabrous dorsally, the ultimate prefemur with one rows of ventrolateral spines, coxopleural process of leg-bearing segment 21 with a dorsal spine).

In this study we describe a new species, *Otostigmustricarinatus* sp. nov., which belongs to the *politus* group. The phylogenetic status of the new species was validated through the molecular analysis of cytochrome *c* oxidase subunit I (COI), 16S rRNA, and 28S rRNA sequences which were derived from 18 *Otostigmus* species.

## ﻿Materials and methods

### ﻿Species sampling and morphological examination

Material was collected from different provinces in China (Table 1, Fig. 2). These specimens were preserved in 75% ethanol, and genomic DNA was extracted from the leg tissue. An Olympus E–M10 II camera was used to capture live colour patterns. Holotypes and paratypes of the new species were maintained deposited at the Institute of Chinese Materia Medica, China Academy of Chinese Medical Sciences, China (**CMMI**).

**Table 1.** *Otostigmus* species vouchers and GenBank accession numbers.

No.	Species	Voucher	Locality	CO1	16S	28S	References
1	*O.tricarinatus* sp. nov.	CMMI 20210315101	Pingbian, Yunnan, China	OM791803	OM793128	–	This study
2	*O.tricarinatus* sp. nov.	CMMI 20200817001	Maguan, Yunnan, China	OM791806	OM793131	OM793116	This study
3	*O.tricarinatus* sp. nov.	CMMI 20220123101	Libo, Guizhou, China	OM791804	OM793129	OM793114	This study
4	*O.tricarinatus* sp. nov.	CMMI 20200712010	Guiping, Guangxi, China	OM791805	OM793130	OM793115	This study
5	*O.tricarinatus* sp. nov.	CMMI 20200712008	Guiping, Guangxi, China	OM791807	OM793132	OM793117	This study
6	*O.polituspolitus* Karsch, 1881	CMMI 20211007101	Tianjing, China	OM791808	OM793133	OM793118	This study
7	*O.scaber* Porat, 1876	CMMI 20190824012	Fuzhou, Fujian, China	OM791809	OM793134	OM793119	This study
8	*O.aculeatus* Haase, 1887	CMMI 20190321002	Xiamen, Fujian, China	OM791810	OM793135	OM793120	This study
9	*O.politusyunnanensis* Lewis, 2003	CMMI 20210315103	Pingbian, Yunnan, China	OM791812	OM793137	OM793122	This study
10	*O.politusyunnanensis* Lewis, 2003	CMMI 20210318108	Yingpan, Yunnan, China	OM791813	OM793138	OM793123	This study
11	*O.beroni* Lewis, 2001	CMMI 20180715101	Jilong, Xizang, China	OM791814	OM793139	OM793124	This study
12	*O.beroni* Lewis, 2001	CMMI 20200504001	Jilong, Xizang, China	OM791815	OM793140	OM793125	This study
13	*O.voprosus* Schileyko, 1992	CMMI 20191031008	Jingxiu, Guangxi, China	OM791816	OM793141	OM793126	This study
14	*O.voprosus* Schileyko, 1992	CMMI 20200914002	Xishuangbanna, Yunnan, China	OM791817	OM793142	OM793127	This study
15	*O.nudus* Pocock, 1890	CES091037	Kerala, Periyar, India	JX531869	JX531739	JX531809	Joshi and Karanth 2012
16	*O.sulcipes* Verhoeff, 1937	CUMZ 00534	Phra Cave, Ban Namyen, Myanmar	MF167805	MF167738	MF167872	Siriwut et al. 2018
17	*O.sulcipes* Verhoeff, 1937	CUMZ 00533	LaosChina borders, Luang Namtha, Laos	MF167804	MF167737	MF167871	Siriwut et al. 2018
18	*O.sulcipes* Verhoeff, 1937	CUMZ 00532	Chiang Mai, Khrai, Thailand	MF167803	MF167736	MF167870	Siriwut et al. 2018
19	*O.spinosus* Porat, 1876	CUMZ 00553	Khanom, Nakhon Si Thammarat, Thailand	MF167785	MF167718	MF167852	Siriwut et al. 2018
20	*O.spinosus* Porat, 1876	CUMZ 00552	Thung Khai Botanical Garden, Yantakhao, Trang, Thailand	MF167784	MF167717	MF167851	Siriwut et al. 2018
21	*O.spinosus* Porat, 1876	CMUZ 00232	Ban na ka som, Attapue, Laos	MF167783	MF167716	MF167850	Siriwut et al. 2018
22	*O.spinicaudus* Newport, 1844	MCZ DNA104645	Kasserine District, Tunisia	–	KF676472	KF676370	Vahtera et al. 2013
23	*O.scaber* Porat, 1876	CUMZ 00530	Wat Ban Hu, Luang Phrabang, Laos	MF167802	MF167735	MF167869	Siriwut et al. 2018
24	*O.scaber* Porat, 1876	CUMZ 00529	Ban Sop Hun, Mueang Ngoi, Luang Phrabang, Laos	MF167801	MF167734	MF167868	Siriwut et al. 2018
25	*O.rugulosus* Porat, 1876	CUMZ 00537	Phu Phiang, Nan, Thailand	MF167787	MF167720	MF167854	Siriwut et al. 2018
26	*O.rugulosus* Porat, 1876	CUMZ 00536	Sai Yok, Kanchanaburi, Thailand	MF167786	MF167719	MF167853	Siriwut et al. 2018
27	*O.rugulosus* Porat, 1876	CUMZ 00535	Chum Ta Bong, Nakhon Sawan, Thailand	MF167782	MF167715	MF167849	Siriwut et al. 2018
28	*O.ruficeps* Pocock, 1890	CES091342	Kollam, Kerala, India	JX531901	JX531771	JX531822	Joshi and Karanth 2012
29	*O.polituspolitus* Karsch, 1881	MCZ DNA106768	Dengfeng, Henan, China	KF676512	KF676470	KF676368	Vahtera et al. 2012
30	*O.multidens* Haase, 1887	CUMZ 00527	Pan Faen, Mae Taeng, Chiang Mai, Thailand	MF167795	MF167728	MF167862	Siriwut et al. 2018
31	*O.multidens* Haase, 188	CUMZ 00526	Taphome Stone Castle, Siem Reap, Cambodia	MF167793	MF167726	MF167860	Siriwut et al. 2018
32	*O.multidens* Haase, 188	CUMZ 00523	Gua I–Kan, Kelantan, Malaysia	MF167779	MF167712	MF167846	Siriwut et al. 2018
33	*O.multidens* Haase, 188	MCZ DNA106502	Central Province, Papua New Guinea	KF676511	KF676469	KF676367	Vahtera et al. 2013
34	*O.astenus* Kohlrausch,1878	CUMZ 00521	Ban Sop Laos, Huaphan, Laos	MF167800	MF167733	MF167867	Siriwut et al. 2018
35	*O.astenus* Kohlrausch, 1878	CUMZ 00520	Tham Mi Ka Ram, Mae Taeng, Chiang Mai, Thailand	MF167799	MF167732	MF167866	Siriwut et al. 2018
36	*O.astenus* Kohlrausch, 1878	MCZ DNA102463	Fiji/Vanuatu	HM453312	HM453221	HQ402532	Vahtera et al. 2012
37	*O.angusticeps* Pocock, 1898	MCZ DNA106500	Finisterre Mountains, Papua New Guinea	KF676509	–	KF676365	Vahtera et al. 2013
38	*O.aculeatus* Haase, 1887	CUMZ 00519	Ban Pha Wong, Mueang Yommarat, Huaphan, Laos	MF167797	MF167730	MF167864	Siriwut et al. 2018
39	*O.aculeatus* Haase, 1887	CUMZ 00518	Wat Phra–Ong Thom, Siam Riep, Cambodia	MF167796	MF167729	MF167863	Joshi and Karanth 2012
40	*O.caraibicus* Kraepelin, 1903	MCZ DNA105633	Puerto Rico	HQ402549	HQ402498	HQ402533	Vahtera et al. 2012
41	*O.voprosus* Schileyko, 1992	IEBR–Chi 033	Xuan Nha, Son La Province, Vietnam	MN861168	–	–	Vu et al. 2020
42	*O.voprosus* Schileyko, 1992	IEBR–Chi 032	Thuong Tien, Vietnam	MN861166	–	–	Vu et al. 2020
43	‘*O.amballae* Chamberlin, 1913’	IEBR–Chi 036	Me Linh, Vietnam	MN861140	–	–	Vu et al. 2020
44	‘*O.amballae* Chamberlin, 1913’	IEBR–Chi 013	Ta Xu, Vietnam	MN861139	–	–	Vu et al. 2020
45	*O.angusticeps* Pocock, 1898	IZ–130684	Finisterre Mountains, Papua New Guinea	KF676509	–	KF676365	Vahtera et al. 2013
46	*R.lewisi*		Kurinjal, Kudremukh National Park, Chikkamagaluru district, India	MK273239	MK273349	MK273461	Joshi and Edgecombe 2017
47	*D.jangii*	CES08915	Kurinjal, Kudremukh National Park, Chikkamagaluru district, India	JX531843	JX531713	JX531789	Joshi and Karanth 2012
48	*R.lewisi*	CES08920	Tadoli, Kudremukh National Park, Chikkamagaluru district, India	MK273240	MK273350	MK273462	Joshi and Edgecombe 2018
49	*D.jangii*	CES08922	Tadoli, Kudremukh National Park, Chikkamagaluru district, India	JX531845	JX531715	JX531791	Joshi and Karanth 2012
50	*A.crotalus*	MCZ DNA100454	Swaziland	AY288742	AY288720	HM453273	Vahtera et al. 2012
51	*A.grandidieri*	MCZ DNA106771	Tanzania	KF676514	KF676473	KF676371	Vahtera et al. 2013

The morphological terminology follows Bonato et al. (2010). The taxonomic characteristics were observed using an Olympus SZ16 stereomicroscope. Helicon Focus 6.7.1 was used to create a multi-focused montage of pictures. The ArcMap 10.7.1 software tool was used to produce the maps.

Abbreviations: **VL** = ventrolateral, **VM** = ventromedial, **M** = medial, **DM** = dorsomedial, **CS** = corner spine, **T** = tergite, **TT** = tergites, **SS** = sternites, **spm** = specimen, **coll.** = collector, **LBS** = leg bearing segment.

### ﻿DNA extraction and fragment amplification

Legs from each specimen were used to extract genomic DNA by using a Promega Wizard SV Genomic DNA Purification Kit (Promega, USA). Polymerase chain reaction (PCR) was used to amplify the cytochrome *c* oxidase subunit I (COI), mitochondrial ribosomal gene 16S, and nuclear ribosomal DNA 28S fragments. Table 2 lists the PCR primers and programs used.

**Table 2.** Primers and programs of PCR.

Loci	Primers Sequence 5’– 3’	Program	Refences
CO1	LCO1490 GGTCAACAAATCATAAAGATATTGG	5 min at 95 °C; 38 cycles of 20s at 95 °C, 20s at 45 °C and 1 min at 72 °C; 3 min at 72 °C	Folmer et al. 1994
	Hcoutout GTAAATATATGRTGDGCTC		
CO1	LCO1490 GGTCAACAAATCATAAAGATATTGG	2 min at 94 °C; 35 cycles of 15s at 95 °C, 40s at 45–47 °C and 15s at 72 °C; 10 min at 72 °C	Folmer et al. 1994; Joshi and Karanth 2011
	Hco2198 TAAACTTCAGGGTGACCAAAAAATCA		
16S	16Sar CGCCTGTTTATCAAAAACAT	5 min at 95 °C; 35 cycles of 30s at 95 °C, 30s at 55 °C and 1 min at 72 °C; 3 min at 72 °C	Xiong and Kocher 1991
	16Sb CTCCGGTTTGAACTCAGATC		
28S	Chilo28SF1 AGCCCAAGTCCCCCTGACC	3 min at 95 °C; 35 cycles of 30s at 95 °C, 30s at 65 °C and 1 min at 72 °C; 3 min at 72 °C	This study
	Chilo28SR1 TATACTCAGGTCCGACGATCGATT		
28S	28Sa GAC CCG TCT TGA AAC ACG GA	2 min at 94 °C; 35 cycles of 15s at 95 °C, 40s at 52 °C and 15s at 72 °C; 10 min at 72 °C	Michael et al. 1997; Joshi and Karanth 2011
	28Sb TCG GAA GGA ACC AGC TAC		

### ﻿Phylogenetic analysis and genetic distance

The genetic distance between *Otostigmus* species was calculated using the Kimura 2-parameter model in MEGA X (Kumar et al. 2018). The COI, 16S, and 28S DNA sequences obtained from this study, as well as previous phylogenetic studies acquired from GenBank (Siriwut et al. 2018; Vu et al. 2020; Joshi and Karanth 2012; Vahtera et al. 2012, 2013), were used as part of the phylogenetic analysis (Table 1). Three partitioned genes, COI, 16S, and 28S, were aligned in BIOEDIT 7.1.3.0 (11/4/2011) (Hall 1999), using the CLUSTAL W tool (Thompson et al. 1994). Bayesian inference (BI) and maximum likelihood (ML) methods were used in the construction of phylogenetic trees. Branch support was evaluated through standard statistical testing (bootstrap support and posterior probability). ML analysis was performed using the IQ-TREE 1.6.8 software tool (Nguyen et al. 2015) in the PHYLOSUITE 1.2.2 platform (Zhang et al. 2020) with 500,000 ultrafast bootstraps (Hoang et al. 2018). MODELFINDER (Kalyaanamoorthy et al. 2017) selected GTR+F+I+G4 as the preferred substitution model. Furthermore, MODELFINDER was used to evaluate the best-fit substitution models of BI, with GTR+F+I+G4 being selected as the optimal substitution model. MRBAYES 3.2.6 (Ronquist et al. 2012) was used to run Bayesian analyses. 10,000,000 generations were used, sampling every 1000 generations, and dividing 25% of the trees as burn-in. A split frequency of less than 0.01 was used to determine stationarity, and a consensus tree was constructed using the remaining trees.

## ﻿Results

### ﻿Order Scolopendromorpha Pocock, 1895

**Family Scolopendridae Leach, 1814**

**Subfamily Otostigminae Kraepelin, 1903**

**Genus *Otostigmus* Porat, 1876**

#### ﻿ Subgenus Otostigmus

Taxon classificationAnimaliaScolopendromorphaScolopendridae

Porat, 1876

A65DB432-4CF6-5C7A-BD84-BEA88938A13D

##### Type species.

*Otostigmuscarinatus* Porat, 1876, by subsequent designation (Pocock 1891: 229).

##### Remarks.

Lewis (2010) counted 58 species in the subgenus Otostigmus. Niu et al. (2021) described *O.xizangensis* from China; Vu et al. (2022) described *O.consonensis* Vu, 2022 from Vietnam; Liu et al. (2022) revalidated O. (O.) lewisi. The subgenus Otostigmus now comprises 62 species and is the largest subgenus in *Otostigmus*.

#### ﻿ Otostigmus politus politus

Taxon classificationAnimaliaScolopendromorphaScolopendridae

Karsch, 1881

D10165C8-3486-5EC4-A326-910D3772BD11

##### Material examined.

**China: Beijing: Haidian District**: Xiangshan Park, 1 spm, CMMI 20210403149, 39.9961°N, 116.1950°E, 370 m asl., 03 April 2021, coll. Tianyun Chen and Zhidong Wang, Fengtai District: 1 spm, CMMI 20190610002, 39.8380°N, 116.3760°E, 50 m asl., 10 June 2019, coll. Chao Jiang; **Dongcheng District**: Beimencang Hutong, 1 spm, CMMI 20190428001, 39.39°N, 116.42°E, 50 m asl., 28 April 2019, coll. Chao Jiang. **Henan Province: Queshan County**: 1 spm, CMMI 20210427101, 32.80°N, 114.02°E, 27 April 2021, coll. Jiong Chen. **Hubei Province: Shiyan**: Wudangshan Mountain, 1 spm, CMMI 20210416111, 32.4307°N, 111.0292°E, 670 m asl., 16 April 2021, coll. Tianyun Chen and Zhidong Wang; **Jingmen**: Dongbao District, Shengjingshan Mountain, 1 spm, CMMI 20210411115, 31.1215°N, 112.1365°E, 470 m asl., 11 April 2021, coll. Tianyun Chen and Zhidong Wang; **Jingshan**: Huzhua Mountain, 1 spm, CMMI 20210410114 31.0774°N, 112.8928°E, 200 m asl., 03 April 2021, coll. Tianyun Chen and Zhidong Wang, Kongshandong Cave, 1 spm, CMMI 20210409110, 30.9735°N, 113.0377°E, 190 m asl., 09 April 2021, coll. Tianyun Chen and Zhidong Wang; **Xiangyang**: Xiangzhou District, Lumensi National Forest Park, 1 spm, CMMI 20190330007, 31.9103°N, 112.2564°E, 130 m asl., 30 March 2019, coll. Chao Jiang. **Jiangsu Province: Yancheng**: Tinghu District, Yancheng National Rare Birds Nature Reserve, 1 spm, CMMI 20201107168, 33.6035°N, 120.5042°E, 0 m asl., 07 Nov. 2020, coll. Chao Jiang; **Lianyungang**: Haizhou District, 1 spm, CMMI 20201105125, 34.5988°N, 119.1703°E, 30 m asl., 05 Nov. 2020, coll. Zhidong Wang; **Nanjing**: Fangshan Mountain, 1 spm, CMMI 20200825106, 31.8953°N, 118.8760°E, 120 m asl., 26 Aug. 2020, coll. Chao Jiang. **Shaanxi Province: Zhashui County**, Dongshan Forest Park, 1 spm, CMMI 20200903111, 33.6811°N, 109.1090°E, 830 m asl., 03 Sept. 2020, coll. Chao Jiang; **Xi’an**: Xi’an Qinling Wildlife Park, 1 spm, CMMI 20191002005, 34.0493°N, 108.8627°E, 10 October 2019, coll. Chao Jiang, Yanta District: Qingliang Mountain Forest Park, 1 spm, CMMI 20190906016, 34.1765°N, 108.9267°E, 420 m asl., 06 Sept. 2019, coll. Chao Jiang. **Shandong Province: Yantai**: Zhifu District: Nanhuansanli, 1 spm, CMMI 20190513004, 37.53°N, 121.41°E, 80 m asl., 13 May 2019, coll. Chao Jiang. **Hebei Province: Yi County**: Yishuihu Lake, 1 spm, CMMI 20190426001, 39.26°N, 115.22°E, 190 m asl., 26 April 2019, coll. Chao Jiang; **Hengshui**: Taocheng District, Qianjin Street, 1 spm, CMMI 20190923002, 37.7511°N, 115.6549°E, 10 m asl., 23 Sept. 2019 coll. Chao Jiang. **Anhui Province: Huaibei**: 1 spm, CMMI 20190418022, 33.9563°N, 116.7984°E, 18 April 2019, coll. Junduo Zhang. **Liaoning Province: Chaoyang**: Shuangta District: Fenghuangshan Mountain, 1 spm, CMMI 20210908101, 41.5604°N, 120.4787°E, 200 m asl., 08 Sept. 2019, coll. Chao Jiang; **Dalian**: 1 spm, CMMI 20190418024, 38.9140°N, 121.6148°E, 18 April 2019, coll. Junduo Zhang. **Tianjin: Nankai District**: Nancuiping Park, 1 spm, CMMI 20211007101, 39.0738°N, 117.1483°E, 0 m asl., 07 October 2021, coll. Chao Jiang. **Gansu Province: Dingxi**: 1 spm, CMMI 20220123104, 35.6080°N, 104.5923°E, 23 Jan. 2022, coll. Quanyu Ji.

#### ﻿ Otostigmus politus yunnanensis

Taxon classificationAnimaliaScolopendromorphaScolopendridae

Lewis, 2003

B9550F37-1CC0-557B-9121-9F1FCB335EE3

##### Material examined.

**China: Yunnan Province: Pingbian Miao Autonomous County**: back mountain of Pingbian Memorial Park, 3 spms, CMMI20210315102–104, 22.9884°N, 103.6931°E, 1380 m asl., 15 March 2021, coll. Chao Jiang, Pingbian Railway Station, 2 spms, CMMI 20210601110 and CMMI 20210601112, 23.0167°N, 103.6353°E, 1010 m asl., 01 June 2021, coll. Chao Jiang; **Jinping Miao, Yao & Dai Autonomous County**: Yingpan township, 4 spms CMMI 20210318105–108, 22.8845°N, 102.9319°E, 1435.40 m asl., 18 March 2021, coll. Chao Jiang.

#### ﻿ Otostigmus tricarinatus sp. nov.

Taxon classificationAnimaliaScolopendromorphaScolopendridae

E3D091B9-AD26-5862-916F-610FD6B2F7A5

https://zoobank.org/EE4ADEE9-2119-4729-87B0-AE4919CF6514

**Figure 1.**
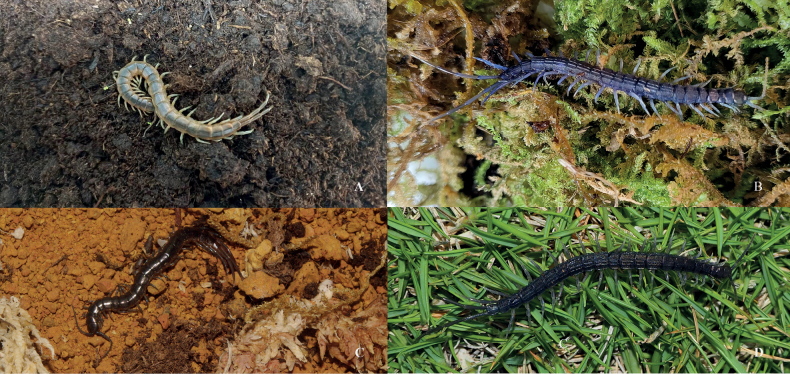
Live specimens of *Otostigmus* spp. from China **A***O.p.politus***B***O.tricarinatus* sp. nov. (Guangxi population) **C***O.p.yunnanensis***D***O.tricarinatus* sp. nov. (Yunnan-Guizhou plateau population).

Figs 1B, D , 3 , 4 Chinese name 三棱耳孔蜈蚣

##### Material examined.

***Holotype*.**CMMI 20210316103, **China: Guangxi Zhuang Autonomous Region: Guiping**: Xishan Town, 23.1147°N, 109.5947°E, 16 March 2021, coll. Mengxuan Shi.

***Paratypes*.** 1 spm, CMMI 20210316161, same data as holotype; 4 spms CMMI 20200712008–011, same data as holotype, 12 July 2020; 1 spm, CMMI 20220327101, **China: Guangxi Zhuang Autonomous Region: Nandan County**, 27 March 2022, coll. Yongxiao Luo. 3 spms, CMMI 20220327102–104; **Hezhou**: Babu District, 24.3751°N, 111.9739°E, 27 March 2022, coll. Xusheng Zhou. **Yunnan Province: Maguan county**, 1 spm, CMMI 20200817001, 23.01°N, 104.39°E, 17 Aug. 2020, coll. Yanan Li. **Pingbian Miao autonomous county**, 1 spm, CMMI 20210315101, back mountain of Pingbian Memorial Park, 22.9884°N, 103.6931°E, 1380 m asl., 15 March 2021, coll. Chao Jiang. **China: Guizhou Province: Libo County**: Jiaou Township, 1 spm, CMMI 20220123101, 25.3017°N, 107.6708°E, 23 Jan. 2022, coll. Quanyu Ji.

##### Etymology.

The name refers to the characteristics of the tergites. The *tri*- compounded with the Latin *carinatus* refers to the three sharp keels on the tergites.

##### Diagnosis.

Antennae with 17 articles, basal three glabrous dorsally, the apical article with a well-developed lateral depression. TT 3–20 with three longitudinal keels. SS 2–19(20) with paramedian sutures occupying anterior 20–100% of sternites, a median depression, and two posterolateral depressions. Coxopleural process with 1–3 apical spines and none or one lateral spine, pore-free median longitudinal strip in pore field from the posterior of sternite 21 to the end of coxopleural process. The ultimate leg prefemur typically with 0–7 spines, lacking corner spine.

**Figure 2.**
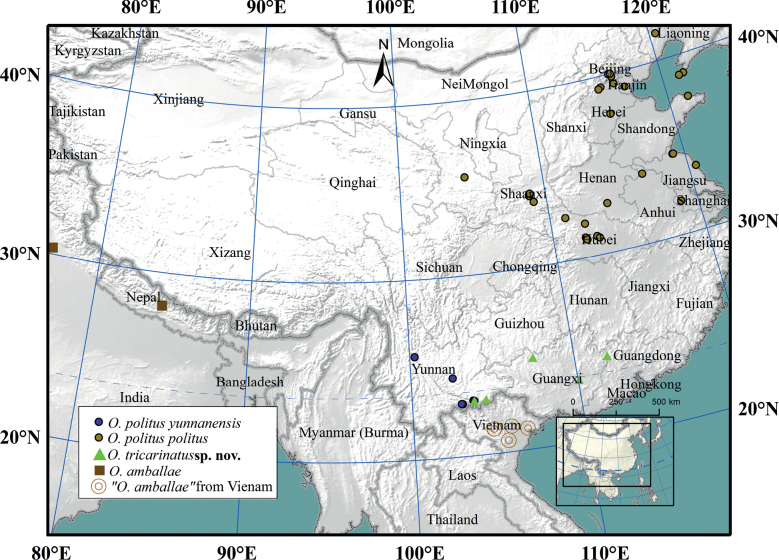
Distribution of *O.politus* species, *O.tricarinatus* sp. nov., and *O.amballae* s.l. in China and Vietnam.

##### Holotype (CMMI 20210316103) description.

Body length 26 mm. Antennae and anterior 1/2 of the cephalic plate, tergites and legs have blue colouration; posterior 1/2 of the cephalic plate, forcipule segment, and sternites have yellow colouration.

**Figure 3.**
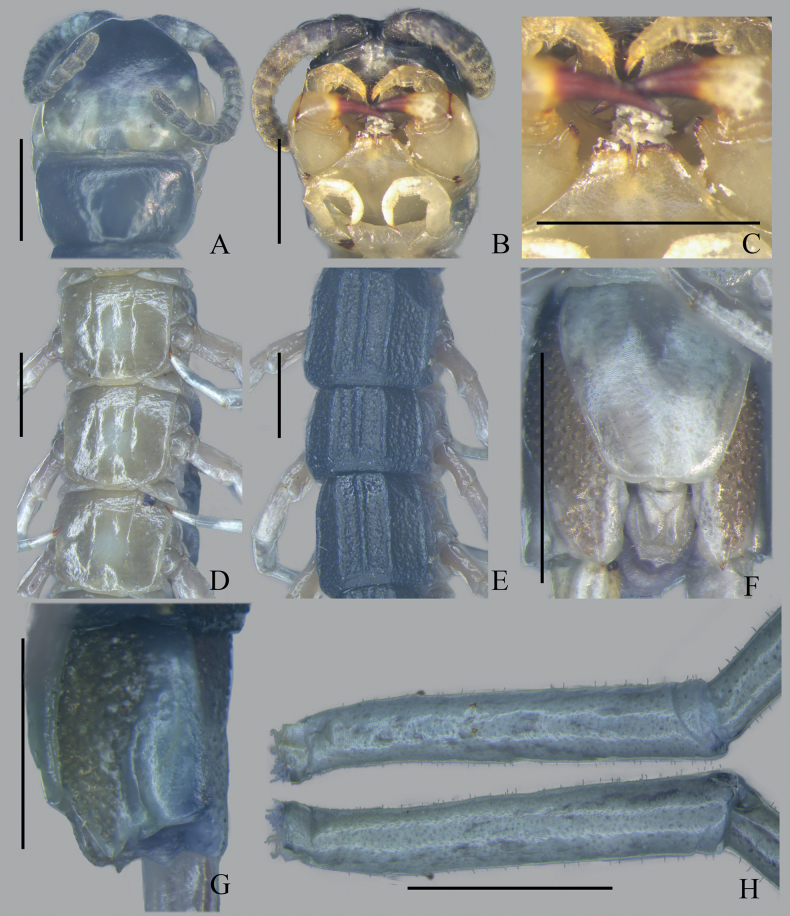
*Otostigmustricarinatus* sp. nov. (Guangxi population) CMMI 20210316103 (HOLOTYPE) **A** cephalic plate (dorsal view) **B** forcipular segment and sternite 1 (ventral view) **C** Forcipular tooth-plates (ventral view) **D**SS 12–14 (ventral view) **E**TT 10–12 (dorsal view) **F**LBS 21 (ventral view) **G**LBS 21 (lateral view) **H** Prefemur of left ultimate leg. Scale bars: 1 mm.

Antennae with 17 articles, 3 glabrous dorsally, 2.5 glabrous ventrally, apical article double the length of the penultimate, with a well-developed lateral depression (Fig. 3A). Antennae reach the posterior margin of T2 when reflexed. Forcipular coxosternite slightly wider than long and lacking sutures/sulci. Coxosternal tooth-plates wider than long, with four teeth. Trochanteroprefemoral process bears one apical and one lateral tubercle.

T1 lacks sutures and convex granules, T2 has sparse convex granules, TT3–21 has scattered convex granules. Tergites without paramedian sutures, TT3–20 with three sharp longitudinal keels, T21 with three incomplete longitudinal keels. Lateral margination at TT3–21. SS2–20 with paramedian sutures occupying anterior 80–100% of sternites (Fig. 3D).

The left coxopleural process has one apical and one lateral spine and the right one has two apical and one lateral spine (Fig. 3F, G). Pore-free median longitudinal strip in pore field from the posterior of sternite 21 to the end of coxopleural process. The ultimate legs are long and slender, with the left prefemur having one ventrolateral and one ventromedial spine. Right prefemur with one ventrolateral spine, one ventromedial spine, and one dorsomedial spine. Ultimate leg relatively long with dense setae (Fig. 3H). Dorsal surface of ultimate prefemur with convex granules, lacking corner spine. Legs 1–5 with two and legs 6–20 with one tarsal spur. Legs 1 and 2 with one tibial spur: leg 1 with one prefemoral and one femoral spur.

##### Variation in paratypes.

Body length 16–50 mm (maximum in CMMI 20220123101). Cephalic plate and T1(2) lacking sutures and convex granules, antennae reached posterior margin of T2–3. Antennae only display 2.5 articles that are glabrous dorsally in the specimen CMMI 20220123101.

Lateral margination at TT 2(3)-21. SS2–20 with paramedian sutures occupying anterior 20–100% of sternites. Coxopleural process with 1–3 apical spines, 0–1 lateral spine and lacking a dorsal spine. Ultimate leg prefemur with 0–7 spines: VL 0–1, M 0–3, VM 1–3, DM 1–2, lacking corner spine. Legs 1–3(4 or 5) typically with two tarsal spurs, subsequent legs to 20, with one tarsal spur.

##### Remarks.

Schileyko (1995) described “*O.amballae* Chamberlin, 1913” on specimens from Vietnam. The morphology of these specimens is different from that of the holotype of *O.amballae* (see Lewis 2002) and were found to be identical to *O.tricarinatus* sp. nov. that has three well-developed longitudinal keels in TT3–20, a very short coxopleural process with no dorsal spine, an ultimate prefemur with two ventral spines, and lacking a corner spine. The holotype of *O.amballae* possesses a low median keel at TT3–20, paramedian sutures each in a sulcus from approximately T13, with two lateral keels on each side of sutures; the coxopleural process is moderately long with a single dorsal spine; the ultimate prefemur with VL3, M3, VM2, DM2, and one corner spine. Lewis (2002) further noted that all specimens assigned to *O.amballae* by Schileyko (1995) should be reassessed.

**Figure 4.**
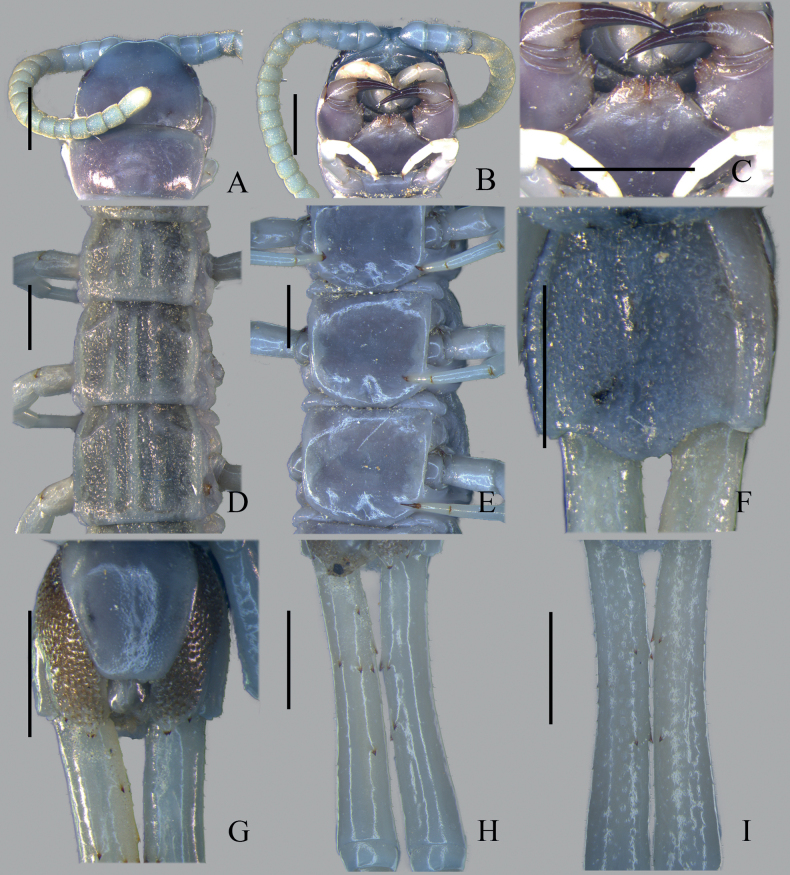
*Otostigmustricarinatus* sp. nov. (Yunnan-Guizhou plateau population) CMMI 20210315101 **A** cephalic plate (dorsal view) **B** forcipular segment and sternite 1 (ventral view) **C** Coxosternal tooth-plates (ventral view) **D**SS 3–5 (dorsal view) **E** Tergites 7–9 (ventral view) **F** Tergite 21 **G** Sternite 21 and coxopleural process (ventral view) **H** Prefemur of ultimate leg (ventral view); **I** Prefemur of ultimate leg (dorsal view). Scale bars: 1 mm.

The material here assigned to *O.tricarinatus* sp. nov. refers to two geographically separate groups: the Yunnan-Guizhou plateau population (Fig. 4) and the Guangxi population (Fig. 3). They are different in the following characteristics: 1) 80% paramedian sutures on sternite in the former population compared to 20% paramedian sutures on sternite in the latter; 2) the ultimate leg prefemur with 4–7 spines in the former population compared to 1–4 spines in the latter; 3) the pore-free median longitudinal strip in pore field absent in the former population while found from the posterior of sternite 21 to the end of coxopleural process in the latter population. Furthermore, phylogenetic analysis revealed that Guangxi population is a sister group to *O.politus* congeners + Yunnan-Guizhou plateau population and has strong node support from both ML and BI analyses (PP = 1, BS = 97%). They are considered to be the same species because no more reliable identification characteristics could find.

##### Distribution.

Fig. 2. China: Guangxi Zhuang autonomous region, Yunnan Province, Guizhou Province; Vietnam (Schileyko 1992, 1995, 2007; Vu et al. 2020).

##### Phylogenetic analysis.

We obtained sequences consisting of 658–812 bp COI, 408–423 bp 16S rRNA, and 368–886 bp28S rRNA. The average K2P genetic distance is 20.1% between *Otostigmus* species, and the range of the K2P distance between *Otostigmus* species is 17.2% (*O.voprosus* against *O.astenus* to 23.1% (*O.politusyunnanensis* against *O.beroni*) (Table 3). The intraspecific divergence among *Otostigmus* taxa varied from 0% to 18.2% (*O.aculeatus*). The mean distance within *O.tricarinatus* sp. nov. was 16.0%(Table 4), which is relatively high among *Otostigmus* species. The interspecific divergence between *O.tricarinatus* sp. nov. and the other *Otostigmus* species fell within a range of 18.4% (*O.spinosus*) to 21.9% (*O.angusticeps*). The interspecific divergence between *O.tricarinatus* sp. nov. and *O.politusyunnanensis* (21.1%) was higher than *O.tricarinatus* sp. nov. and *O.polituspolitus* (19.3%). The high genetic distances found within the *Otostigmus* species imply that they may have been misidentified or contain cryptic species. Therefore, species delimitation of *Otostigmus* requires further study with additional samples as well as genetic data from various populations.

**Table 3.** Mean K2P genetic distance between the *politus* group, *aculeatus* group and *rugulosus* group species based on COI sequences.

	(1)	(2)	(3)	(4)	(5)	(6)	(7)	(8)	(9)
*O.angusticeps* (1)									
*O.aculeatus* (2)	22.2%								
*O.astenus* (3)	18.3%	22.9%							
*O.beroni* (4)	21.8%	23.9%	17.7%						
*O.polituspolitus* (5)	21.1%	20.7%	20.5%	20.2%					
*O.politusyunnanensis* (6)	22.5%	22.5%	21.8%	23.8%	18.5%				
*O.rugulosus* (7)	22.7%	20.1%	20.1%	21.9%	20.0%	20.6%			
*O.spinosus* (8)	19.6%	19.8%	19.3%	19.1%	18.9%	22.0%	18.8%		
*O.tricarinatus* sp. nov. (9)	22.5%	22.4%	20.5%	21.8%	19.3%	21.1%	21.7%	19.0%	
*O.voprosus* (10)	20.8%	22.9%	17.4%	17.5%	22.1%	21.9%	22.4%	20.0%	21.5%

**Table 4.** Mean K2P genetic distance within the *politus* group, *aculeatus* group and *rugulosus* group species based on COI sequences.

Examined species	Mean distance	Standard error
*O.angusticeps*	–	–
*O.aculeatus*	18.2%	1.5%
*O.astenus*	17.5%	1.3%
*O.beroni*	0.2%	0.1%
*O.polituspolitus*	8.4%	1.3%
*O.politusyunnanensis*	16.9%	1.7%
*O.rugulosus*	6.5%	0.9%
*O.spinosus*	15.1%	1.3%
*O.tricarinatus* sp. nov.	16.0%	1.1%
O.voprosus	15.4%	1.2%

**Figure 5.**
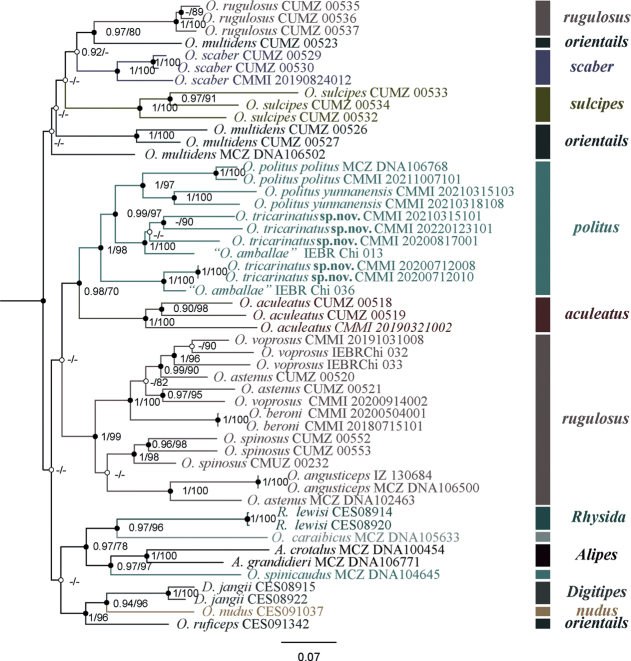
Maximum likelihood phylogenetic tree and Bayesian phylogenetic tree based on combined data for *Otostigmus* along with posterior probability (PP > 0.9 / BS > 70%) values for each node.

Sequences from the new species, as well as 47 other Otostigminae samples from different species groups, were aligned. Included in the alignment were two COI sequences from samples IEBR–Chi 013 and IEBR–Chi 036 from Vietnam, which were misidentified as “*O.amballae* Chamberlin, 1913” (Vu et al. 2020). ML and BI analyses were utilised to construct phylogenetic trees for the combined COI+16S+28S dataset. Our results moderately supported the *politus* group, *aculeatus* group, and species of the *rugulosus* group, from both ML and BI analyses (BS > 70% and PP > 0.9) (Fig. 5). The reciprocally monophyletic *politus* group and *aculeatus* group were found to have moderate levels of support (PP = 1; BS = 71%). In this study, *O.tricarinatus* sp. nov. was assigned to the *politus* group. *Otostigmustricarinatus* sp. nov. (Yunnan-Guizhou plateau population) and one sample from Vietnam: Me Linh (IEBR–Chi 013) was determined to be sister to *O.polituspolitus* and *O.politusyunnanensis*, with strong node support (PP = 1 and BS = 97%). *Otostigmustricarinatus* sp. nov. (Guangxi population) together with one sample from Vietnam, Ta Xua (IEBR–Chi 036), was observed as having high levels of support for being sister to the clade of *O.polituspolitus* + *O.politusyunnanensis* + *O.tricarinatus* sp. nov. (Yunnan-Guizhou plateau population) in both BI and ML analyses (PP = 1, BS = 97%).

At last, the key for *Otostigmus* species in China is provided.

### ﻿Key to species of the *Otostigmus* in China

**Table:** 

1	The ultimate leg prefemur with two rows of ventrolateral spines	***O.aculeatus***
–	The ultimate leg prefemur with one row of ventrolateral spines	**2**
2	Antennae with 19–22 articles	**3**
–	Antennae with 17 or 18 articles	**5**
3	Tooth-plates with 3+3 teeth; tergites without keels	***O.astenus***
–	Tooth-plates with 4+4 teeth; tergites with keels	**4**
4	TT5–20 with a median keel, coxopleural process curved in females	***O.xizangensis***
–	TT3(5)–20 with 7–9 longitudinal keels, coxopleural without curved in females	***O.scaber***
5	Basal 3 antennal articles glabrous dorsally	**6**
–	Basal 2–2.75 antennal articles glabrous dorsally	**10**
6	Tooth-plates with 3+3 teeth, coxopleural process with a dorsal spine	***O.voprosus***
–	Tooth-plates with 4+4 teeth coxopleural process without a dorsal spine	**7**
7	Ultimate prefemur with 9–16 spines; with corner spine	**8**
–	Ultimate prefemur with 0–7 spines; without corner spine	***O.tricarinatus* sp. nov.**
8	Ultimate prefemur with 9–12 spines (3–4VL, 2–3VM, 2–3M, 1–1DM)	***O.polituspolitus***
–	Ultimate prefemur with 15–16 spines (5VL, 4–5VM, 4M, 2DM)	***O.politusyunnanensis***
9	Coxopleural process with 1 apical spine	***O.martensi***
–	Coxopleural process with 2 apical spines	**10**
10	Legs 1–4 or 5 with tibial spur, 2 tarsal spurs on legs 1–16(19)	***O.beroni***
–	Legs 1–2 with tibial spur, the distribution of 2 tarsal spurs in legs without regularity	***O.lewisi***

## ﻿Discussion

Vu et al. (2020) employed a 638 bp COI loci for phylogenetic analysis of Vietnam *Otostigmus* centipedes and discovered that the “*O.amballae*” from Vietnam was not monophyletic. We made use of three loci, consisting of COI, 16S, and 28S rRNA, to construct a phylogenetic tree, and verified that the Vietnamese “*O.amballae*” specimens have sufficient support to form two clades: specimen IEBR–Chi 013 (Vietnam, Ta Xua) form a clade with Guangxi population of the newly discovered species *O.tricarinatus* sp. nov. and specimen IEBR–Chi 036 (Vietnam, Me Linh) forms another clade with *O.tricarinatus* sp. nov. (Yunnan-Guizhou plateau population). The morphological characteristics of *O.tricarinatus* sp. nov. is different from the holotype of *O.amballae* (Lewis 2002) in terms of keels on the tergite, a corner spine of the ultimate leg prefemur, and a dorsal spine in the coxopleural process. Combined with the phylogenetic results, we propose that all Vietnamese specimens previously assigned to *O.amballae* Chamberlin, 1913 must be referred to *O.tricarinatus* sp. nov.

The presence of three well-developed longitudinal keels on TT3–20 is the main morphological character that distinguishes *O.tricarinatus* sp. nov. from other Otostigmus (Otostigmus) species. The keels on tergites are a widespread morphological characteristic in Otostigminae Kraepelin, 1903, and may be found in the genera/subgenera *Alipes*, *Edentistoma*, Otostigmus (Parotostigmus) and O. (Otostigmus). *Otostigmusscaber*, *O.rugulosus* Porat, 1876, and *O.amballae* generally have tergal longitudinal keels (Schileyko 1995; Lewis 2002, 2010; Song et al. 2005). *Otostigmusastenus* Kohlrausch, 1878 (Lewis 2002), *O.geophilinus* Haase, 1887, *O.cuneiventris* Porat, 1893 (Lewis 2015) and *O.angusticeps* Pocock, 1898 (Lewis 2001, 2014) often have low tergal keels; *O.polituspolitus* (Beijing, Tianjin), *O.politusyunnanensis* (Yunnan) and *O.reservatus* Schileyko, 1995 (Hainan) have a low longitudinal median keel. The following characters distinguish the new species from *O.scaber* and *O.rugulosus*: antennal articles (17 in *O.tricarinatus* sp. nov. 20–21 in *O.scaber*, 19–21 in *O.rugulosus*); tergal paramedian sutures (without tergal paramedian sutures in *O.tricarinatus* sp. nov., TT5(6)–20 with paramedian sutures in *O.scaber* and TT4(5)–20 with tergal paramedian sutures in *O.rugulosus*); tergal longitudinal keels (T3–20 with 3 longitudinal keels in *O.tricarinatus* sp. nov., TT3(5)–20 with 5–7(9) longitudinal keels in *O.scaber*, low median ridge or keel from 3(5), low rounded lateral keels on posterior segments 7–17(10–14) in *O.rugulosus*); coxopleural process spines (*O.tricarinatus* sp. nov. without a dorsal spine, *O.scaber* and *O.rugulosus* with a dorsal spine); coxopleural pore-free median longitudinal strip in pore field (absent in Yunnan-Guizhou plateau population of *O.tricarinatus* sp. nov., extending 50–75% length in *O.scaber*, 100% length from the posterior of sternite 21 to the end of coxopleural process in Guangxi population of *O.tricarinatus* sp. nov. and *O.rugulosus*); legs with tarsal spines (1–3(4\5) with 2, the subsequent to 20 with 1 in *O.tricarinatus* sp. nov., 1–9(7\8\10\11) with 2, the subsequent to 19 or 20 with one in *O.scaber*, 1–11(up to 18) with 2, the subsequent to19 with 1 in *O.rugulosus*); corner spine (without corner spine in *O.tricarinatus* sp. nov., with corner spine in *O.scaber* and *O.rugulosus*). Table 5 presents the morphological characters of *O.tricarinatus* sp. nov., *O.scaber*, *O.amballae*, and *O.rugulosus*.

**Table 5.** Comparison of four species with keels (**1**Schileyko 1995; **2**Lewis 2002; **3**Song et al. 2005; **4**Lewis 2010; **5** this study).

Characteristics	*O.scaber* Porat, 1876 (1–3)	*O.tricarinatus* sp. nov. (5)	*O.rugulosus* Porat, 1876 (4)	*O.amballae* Chamberlin, 1913 (2)
Length(mm)	14–69	16–50	32–43	38
Antennal articles	20–21	17	19–21	17
Basal antennal articles glabrous	2–2.5	3 dorsally, 2.5 ventrally	2–2.25(2.4)	2.5
Lateral depression on apical antennal article	absent	present	absent	present
Teeth on coxosternal tooth-plates	4–5	4	4	4
Teeth on the trochanteroprefemoral process	2–4	2	2	2
Tergite paramedian sutures from	5(6)–20	without	4(5)	Complete from tergite 3
Tergite longitudinal keels	3(5)–20 with 5–7(9) longitudinal sutures	T3–20 with 3 longitudinal keels	low median ridge or keel from 3(5), low rounded lateral keels on posterior segments 7–17(10–14)	A low median keel from 3–20, paramedian sutures forming two lateral keels on each side from ca. 13
Tergites marginate starting from	5–7	2 or 3	9(7)	6
Sternal paramedian sutures/sulci	3–19, incomplete sutures	4–19 with 20–80% sutures on anterior part of sternites	30–55% of mid and posterior sternites 55% on posterior sternites	2–20 with low tubercles, 14–20 with round median depression
Coxopleural process spines	AP2–3, LP1–3, DP1	AP1–3, LP 0–1	AP2, SAP1–3 LP1, DP1	AP2, LAP1, DP1–2
pore-free median longitudinal strip in pore field	extending 50–75% length from the posterior of sternite 21 to the end of coxopleural process	from the posterior of sternite 21 to the end of coxopleural process	from the posterior of sternite 21 to the end of coxopleural process	from the posterior of sternite 21 to the end of coxopleural process
Legs with tarsal spurs	1–9(7, 8, 10, 11) with 2, the subsequent to 19 or 20 with one	1–3(4, 5) with 2, the remainder with 1, 21 without	1–11(up to 18) with 2, 20–21 without	Many legs missing. Legs 1 and 2 with two tarsal spines, legs 10 and 13–20 with one
Spines of ultimate prefemur	VL3–4(5), VM2–3, DM2–3	VL0–1, M0–3, VM0–3, DM0–2	3–4 rows of VL4, M0–3, M1–2, DM0–2	VL3, M3, VM2, DM2
Corner spine	1	0	1	1

Phylogenetic analysis revealed that *O.tricarinatus* sp. nov., *O.polituspolitus*, *O.politusyunnanensis* formed a clade constituting the *politus* group. *Otostigmuspolituspolitus* and *O.politusyunnanensis* are reciprocally monophyletic and form a clade which is sister to *O.tricarinatus* sp. nov. (Yunnan-Guizhou plateau population). Furthermore, *O.tricarinatus* sp. nov. (Guangxi population) is a sister clade of *O.tricarinatus* sp. nov. (Yunnan-Guizhou plateau population), *O.polituspolitus*, and *O.politusyunnanensis*. However, *O.polituspolitus*, distributed in North China and Korea, is geographically isolated from other *politus*-group members. The records of *O.polituspolitus* from Vietnam with a long ultimate prefemur and spine arrangement were 5–6VL, 5 VM, and 3DM (Schileyko 2007), which resembles *O.politusyunnanensis* rather than *O.polituspolitus*.

## Supplementary Material

XML Treatment for Subgenus Otostigmus

XML Treatment for Otostigmus politus politus

XML Treatment for Otostigmus politus yunnanensis

XML Treatment for Otostigmus tricarinatus

## ﻿Additional information

### Author ORCIDs

Tian-Yun Chen https://orcid.org/0000-0001-9761-0996

Chao Jiang https://orcid.org/0000-0003-1841-1169

### Data availability

All of the data that support the findings of this study are available in the main text or Supplementary Information.
